# Next Generation Sequencing for HIV-1 Drug Resistance Testing—A Special Issue Walkthrough

**DOI:** 10.3390/v13020340

**Published:** 2021-02-22

**Authors:** Rami Kantor

**Affiliations:** Division of Infectious Diseases, Alpert Medical School, Brown University, Providence, RI 02906, USA; rkantor@brown.edu

Drug resistance remains a global challenge in the fight against the HIV pandemic [1,2]. Where feasible, guidelines recommend testing for HIV drug resistance before initiation of and upon inadequate response to antiretroviral therapy in order to guide regimen selection [3,4]. Where less available, such testing is recommended only in specific populations and circumstances [5], and where even less accessible, it is recommended only for public health surveillance [6]. Sanger sequencing, available since the 1970s [7], has been the conventional technology used for HIV drug resistance testing [3,8]. Essential external quality assurance (EQA) strategies, needed to ensure reliable results of complex Sanger sequencing-based HIV drug resistance testing assays, have supported laboratories for decades [9,10,11,12]. More recently introduced next generation sequencing (NGS) technologies are increasingly used in diverse circumstances, including for HIV drug resistance testing [13,14]. Whether these technologies can and should replace Sanger sequencing for HIV drug resistance testing, and if so, in which settings and circumstances, is unclear. In that context, validated EQA strategies to support laboratories using NGS for HIV drug resistance testing are essential, due to more complex NGS-based methods, yet such strategies remain to be established. Developing such EQA strategies, as well as more standardized laboratory- and bioinformatics-related considerations, are important early steps towards widespread implementation of NGS for HIV drug resistance testing [15,16,17].

In February 2018, an international symposium on bioinformatic strategies for NGS-based HIV drug resistance testing was held in Winnipeg, Canada. Outcomes of the symposium included proposed standardizations of NGS data processing, quality control, and reporting and management strategies for HIV drug resistance testing. The intent was for these standardizations to serve as a starting guideline for NGS HIV drug resistance data processing that informs the refinement of existing pipelines and those yet to be developed [18].

In September 2019, the Second International Symposium on NGS HIV Drug Resistance was held in Winnipeg, Canada, to focus on EQA strategies for NGS-based HIV drug resistance testing. Symposium deliberations emphasized logistical and implementation needs and considerations, clarified existing gaps, and helped with the identification of public health and programmatic resolutions. This Special Issue was assembled and designed to allow symposium participants to highlight these discussions, and to enable readers to learn and think about this important topic and consider ways forward. This Editorial provides a brief walkthrough of the Special Issue’s 10 manuscripts, each of which stands on its own, yet they all address the theme of the aforementioned symposium and of the supplement. The overall design of this Special Issue includes an overview of the topic of NGS for HIV-1 drug resistance testing, lessons from the past and how they can guide us, special considerations, preliminary actual data, and logistical concerns towards the future.

Ji et al. [19] and Avila-Rios et al. [20] provide an overview to the theme of the Special Issue. Ji et al. introduce the Second Winnipeg Symposium and highlight existing technical and knowledge gaps related to the adoption of NGS for HIV-1 drug resistance testing in clinical care, public health, and research. Avila-Rios et al. then focus on laboratory, clinical, and implementation considerations and the need for standardization and quality assurance of NGS-based HIV-1 drug resistance genotyping. Both papers discuss potential sources of variation and bias in the general NGS workflow, with some focus on resource limited settings, and present the need for the establishment of EQA programs to address existing challenges.

The next group of manuscripts present lessons from the past and how they might impact our way forward when considering NGS for HIV-1 drug resistance testing. Jennings et al. [21] discuss challenges in applying experience earned from a Sanger-based EQA strategy within the National Institute of Allergy and Infectious Diseases (NIAID) Virology Quality Assurance (VQA) program towards NGS HIV-1 drug resistance assays, including already started initiatives. Lee et al. [22] then present unique requirements and challenges in conducting EQA for NGS-based HIV-1 drug resistance testing, and consider the differences such a program might mandate as compared to the conventional Sanger sequencing approach.

Special considerations relevant for using NGS to detect HIV-1 drug resistance mutations are discussed in the next group of manuscripts. Zhou et al. [23] focus on the important yet challenging accurate detection of minor drug-resistant variants in HIV-1 quasispecies, the potential for this error-prone process to confound interpretation, and existing ways to remove such errors. Capina et al. [24] then consider the importance of internal laboratory complex NGS quality control processes and how they might challenge conventional quality management operations. Finally, Noguera-Julian et al. [25] discuss ‘dry laboratory data panels’ (rather than ‘wet laboratory sample panels’) and how they can support EQA programs for NGS-based HIV-1 drug resistance testing. Such panels have been used for Sanger sequencing, and are needed, considering that bioinformatic analyses remain an important bottleneck that should be addressed, particularly with the more complex NGS data interpretation process.

The next two manuscripts in this Special Issue present initial data from actual use of an NGS EQA program. Becker et al. [26] discuss performance assessment criteria for NGS-based HIV-1 drug resistance assays and propose a new validation, evaluation, and standardization system that could be used for accreditation and quality assurance purposes. Then Parkin et al. [27] describe a preliminary multi-laboratory comparison of NGS to Sanger sequencing for HIV-1 drug resistance testing, with close attention to the detection of minority drug-resistance variants.

In the final manuscript of the Special Issue, Ji et al. [28] summarize the last session of the symposium, and discuss logistical considerations, which may inform the development of an EQA program for NGS HIV-1 drug resistance testing.

Taken together, I hope that the contents of the Special Issue will contribute to the continued imperative discussion on if and how NGS should be incorporated into HIV-1 drug resistance testing for clinical care, research, and public health, and the unique role of a dedicated EQA program in this process. A ‘consensus’ to address such questions is currently lacking, however, this conversation is valuable and essential towards planning the next steps and establishing EQA programs to support such steps.

Finally, I would like to thank all of the authors, reviewers, editors, and *Viruses* personnel who made this Special Issue possible. Special thanks go to the Organizing Committee of the Second International Winnipeg Symposium on NGS HIV-1 Drug Resistance, headed by Drs. Hezhao Ji and Paul Sandstrom.
